# GenSeed-HMM: A Tool for Progressive Assembly Using Profile HMMs as Seeds and its Application in *Alpavirinae* Viral Discovery from Metagenomic Data

**DOI:** 10.3389/fmicb.2016.00269

**Published:** 2016-03-04

**Authors:** João M. P. Alves, André L. de Oliveira, Tatiana O. M. Sandberg, Jaime L. Moreno-Gallego, Marcelo A. F. de Toledo, Elisabeth M. M. de Moura, Liliane S. Oliveira, Alan M. Durham, Dolores U. Mehnert, Paolo M. de A. Zanotto, Alejandro Reyes, Arthur Gruber

**Affiliations:** ^1^Department of Parasitology, Institute of Biomedical Sciences, University of São PauloSão Paulo, Brazil; ^2^Graduate program in Computational Biology, Universidad de los AndesBogotá, Colombia; ^3^Department of Microbiology, Institute of Biomedical Sciences, University of São PauloSão Paulo, Brazil; ^4^Department of Computer Science, Institute of Mathematics and Statistics, University of São PauloSão Paulo, Brazil; ^5^Department of Biological Sciences, Universidad de los AndesBogotá, Colombia; ^6^Center for Genome Sciences and Systems Biology, Department of Pathology and Immunology, Washington University in Saint LouisMO, USA

**Keywords:** *Alpavirinae*, sequence assembly, metagenomic analysis, viral discovery, *de novo* diagnosis

## Abstract

This work reports the development of GenSeed-HMM, a program that implements seed-driven progressive assembly, an approach to reconstruct specific sequences from unassembled data, starting from short nucleotide or protein seed sequences or profile Hidden Markov Models (HMM). The program can use any one of a number of sequence assemblers. Assembly is performed in multiple steps and relatively few reads are used in each cycle, consequently the program demands low computational resources. As a proof-of-concept and to demonstrate the power of HMM-driven progressive assemblies, GenSeed-HMM was applied to metagenomic datasets in the search for diverse ssDNA bacteriophages from the recently described *Alpavirinae* subfamily. Profile HMMs were built using *Alpavirinae*-specific regions from multiple sequence alignments (MSA) using either the viral protein 1 (VP1; major capsid protein) or VP4 (genome replication initiation protein). These profile HMMs were used by GenSeed-HMM (running Newbler assembler) as seeds to reconstruct viral genomes from sequencing datasets of human fecal samples. All contigs obtained were annotated and taxonomically classified using similarity searches and phylogenetic analyses. The most specific profile HMM seed enabled the reconstruction of 45 partial or complete *Alpavirinae* genomic sequences. A comparison with conventional (global) assembly of the same original dataset, using Newbler in a standalone execution, revealed that GenSeed-HMM outperformed global genomic assembly in several metrics employed. This approach is capable of detecting organisms that have not been used in the construction of the profile HMM, which opens up the possibility of diagnosing novel viruses, without previous specific information, constituting a *de novo* diagnosis. Additional applications include, but are not limited to, the specific assembly of extrachromosomal elements such as plastid and mitochondrial genomes from metagenomic data. Profile HMM seeds can also be used to reconstruct specific protein coding genes for gene diversity studies, and to determine all possible gene variants present in a metagenomic sample. Such surveys could be useful to detect the emergence of drug-resistance variants in sensitive environments such as hospitals and animal production facilities, where antibiotics are regularly used. Finally, GenSeed-HMM can be used as an adjunct for gap closure on assembly finishing projects, by using multiple contig ends as anchored seeds.

## Introduction

From the golden age of phage research establishing the basis for the development of molecular biology, virus research suffered a decline due to several technical difficulties, in particular the necessity of knowing the specific viral and host life cycles and conditions for *in vitro* growth (Rosenberg, 2015). With the advent of next generation sequencing (NGS) and metagenomics, viral discovery and research entered a new successful age. A pioneering metagenome study, a virome of uncultured marine viral communities (Breitbart et al., 2002), revealed a predominance of bacteriophages, and demonstrated the potential of metagenomics in the field of viral research. Since then, viral ecology has risen as a new field, and it is now possible to assess the viral composition of a microbial community and understand the fundamental role that these highly abundant biological entities play in any environment, with particular efforts shown in marine environments (Rohwer and Thurber, 2009). However, since the very start of the metagenomic bloom, it has been clear that our knowledge of viral diversity is scarce and relies on viruses where the host is known and can be cultivated, severely restricting the known viral diversity to possibly less than 1% of what is actually out there (*cf*. Fancello et al., 2012). Furthermore, the rate of shotgun data generation has outpaced the sequencing of reference viral genomes, and this ever-increasing gap limits our capacity to analyze newly generated datasets. Thus, the development of new computational tools is of utmost importance to increase our understanding of viral diversity (Fancello et al., 2012). Some of the most important pandemic diseases arose by the transmission of viruses originally present in animals that were able to adapt to the human host (Wang, 2011; Rosenberg, 2015). Thus, a systematic surveillance for emerging viruses is crucial to enable the detection of novel and potentially devastating ones before they become pandemic (Lipkin and Firth, 2013; Smits and Osterhaus, 2013).

The human and animal microbiome field has benefited immensely from the advances in NGS and metagenomics (Tang and Chiu, 2010; Bexfield and Kellam, 2011). The number of studies characterizing the gut microbiome has increased exponentially in recent years, and such studies have linked changes in these complex communities to diseases ranging from obesity and malnutrition to even Alzheimer's and autism (Mayer et al., 2014). An important component of this microbial community is the viral one, in particular phages that are an integral part of the community (Reyes et al., 2010, 2012, 2015; Dutilh et al., 2014; Norman et al., 2015). Since the early studies of the viral component of the gut microbiota, an important limitation has been the lack of reference viral genomes infecting the Firmicutes and Bacteroidetes, which constitute the most abundant bacterial phyla inhabiting the gut (Arumugam et al., 2011) Bacteriophages are gaining growing relevance in gut microbiome studies where changes in viral and phage population have been linked to alterations in the microbial community and/or human health (Norman et al., 2015; Reyes et al., 2015).

*Alpavirinae*, a recently characterized subfamily of the *Microviridae* family, is composed of ssDNA phages that exist either as temperate phages of Bacteroidetes genomes (Kim et al., 2011; Krupovic and Forterre, 2011) or infectious particles (Roux et al., 2012; Zhong et al., 2015). Roux et al. (2012) analyzed metagenomic data from different geographic locations and biological sources, and described a large set of complete, previously undescribed *Microviridae* genomes, including 33 *Alpavirinae* genomes. More recently, Quaiser et al. (2015) described 17 additional complete *Microviridae* genomes from a *Sphagnum*-dominated peatland. A recent study (Zhong et al., 2015) reported the occurrence of *Microviridae* in peri-alpine lakes, mainly represented by gokushoviruses, but also including *Alpavirinae*, a finding that confirms that this latter group is also present in fresh waters, possibly in both lysogenic and lytic forms. Cantalupo et al. (2011) found diverse viral populations in raw sewage, with 80% of the metagenomic reads being related to bacteriophages and, from this subset, 37% were derived from *Microviridae*. Considering that relatively few genomes of the *Alpavirinae* subfamily have been described so far and their initial description as Bacteroidetes associated viruses, this taxonomic group constitutes an interesting case study for a new viral discovery strategy.

One of the most challenging tasks for metagenomic data analysis is the assembly phase (Wajid and Serpedin, 2012; El-Metwally et al., 2013). Several algorithms have been developed and can roughly be classified according to the graph construction method: greedy, OLC (overlap-layout-consensus), and de Bruijn graphs. Assemblers using the OLC method are most appropriate for datasets of relatively long reads, such as Sanger and 454 platforms, but the quadratic complexity of the overlap computation phase severely limits the size of the datasets that can be used. Assemblers using *k*-mers and de Bruijn graphs require much less computational power, but memory requirement is still high. Therefore, whatever the algorithm, sequence assemblers are highly demanding in terms of memory usage and/or processing power, especially for datasets in the magnitude of millions of reads. Additionally, most *de novo* assemblers have been developed for single-organism genome sequencing (Fancello et al., 2012). In fact, *de novo* assembly of metagenomic data is particularly challenging for several reasons, among others: (1) the heterogeneous nature of the sample, with many different organisms; (2) uneven distribution of organism quantities, leading to biased sampling and coverage; (3) unlike single-organism genome sequencing, the number of final assembled sequences cannot be predicted; (4) sequences derived from closely related organisms may generate chimeric assemblies; (5) polymorphisms, in a way similar to sequencing errors, can disrupt assemblies by tangling the assembly graph (i.e., by creating specific topological structures such as tips and bubbles). With those challenges in mind, a few recent attempts have been made to either modify traditional assemblers or develop assemblers specifically designed for metagenomic data (Fancello et al., 2012). However, such approaches still suffer from the same computational resource drawbacks mentioned above for traditional genome assemblers.

Many sequencing projects do not have as a goal the reconstruction of all possible sequences present in a sample, but rather aim at studying a well-defined gene, gene family, or a transcript. In this case, a target-specific assembly could represent a more sensible approach. To fulfill such a need, our group was the first one to develop a seed-driven progressive assembly algorithm, implemented in the GenSeed program (Sobreira and Gruber, 2008), as a rational method to reconstruct specific targets from unassembled sequence datasets. GenSeed uses a short DNA or protein sequence as a query in similarity searches to select reads, which in turn are retrieved from the dataset and assembled together with the seed sequence, leading to an increment of its original length. Short sequences are then extracted from the assembled sequence ends and used as new seeds in an iterative process that generates progressively longer sequences at each assembly cycle. Because assembly is performed in multiple steps and relatively few reads are used in each cycle, the program demands low computational resources. Some recent approaches based on the same concept of seed-driven iterative assembly have been proposed for the assembly of viral sequences from metagenomic data (Smits et al., 2015), but they are all restricted to the use of DNA sequence seeds. In this work, we report the development of GenSeed-HMM, a completely revised and highly incremented version of GenSeed. The proposed approach relies on two principles: (1) progressive assembly as an alternative for sequence reconstruction; and (2) the use of profile HMMs as starting seeds for target-driven reconstruction. As a proof of principle, we use GenSeed-HMM and profile HMMs built from *Alpavirinae* proteins to reconstruct novel viral sequences from human fecal samples. GenSeed-HMM allowed the reconstruction of many *Alpavirinae* genomes distinguishable from those described by Roux et al. (2012), outperforming conventional (global) genomic assembly in several metrics. GenSeed-HMM provides a fast and simple way to run progressive sequence assembly pipelines that are directly targeted at sequences of interest, potentially detecting members of a taxonomic group related but not equal to those used on the construction of the profile HMM. This feature opens up the possibility of diagnosing novel viruses, without previous specific information.

## Materials and methods

### Data sources

Two distinct metagenomic datasets were used in this study, derived from fecal microbiota and raw sewage samples. The metagenomic sequence data from fecal microbiota was obtained from monozygotic twins and their mothers, and sequenced on the 454 platform, as previously described (Reyes et al., 2010). Sequence datasets (accession codes SRX028823 to SRX028827) were downloaded from the Sequence Read Archive (SRA) at http://www.ncbi.nlm.nih.gov/sra. SRA format files were converted into FASTQ using the *fastq-dump* program (SRA toolkit) and all adaptors were trimmed with *cutadapt* (https://cutadapt.readthedocs.org) using parameters -q 30–minimum-length 50 –overlap = 5 -u 14. Raw sewage (total volume of 15 L) collected at the municipality of Taboão da Serra (São Paulo, Brazil) was pressure-filtered through an AP-20 filter membrane (Merck Millipore) and electropositive filter membranes Zeta Plus 60 (AMF, Cuno Div.). Viruses were then eluted in a protein mix, concentrated by ultracentrifugation and treated with Vertrel XF (decafluoropentane, DuPont) to remove lipids and proteins (Mehnert and Stewien, 1993; Queiroz et al., 2001). Viral DNA was extracted using DNeasy Blood and Tissue kit (Qiagen®) and amplified with an illustra™Single Cell GenomiPhi™DNA Amplification Kit (GE Healthcare Life Sciences). The DNA was used to construct a library with the Nextera XT DNA Library Preparation Kit (Illumina, Inc.) and sequenced using the Illumina HiSeq 2500 System, generating 101-bp paired-end reads. To remove the Nextera transposase sequence, FASTQ files were trimmed with *cutadapt* using parameters -q 30 -a CTGTCTCTTATACACATCT –minimum-length 50 –overlap=5 -u 2.

### GenSeed-HMM development and progressive assembly

GenSeed-HMM was developed in the Perl language and is publicly available for download under the terms of the GNU General Public License version 3 at http://genseedhmm.sourceforge.net. Installation instructions and documentation are also provided. All tests reported in this work were performed on a Dell PowerEdge T710 server with two Intel Xeon X5660 2.8 Ghz processors and 64 GB of RAM. GenSeed-HMM can be used in any POSIX-compliant operating system such as UNIX and Linux distributions with an installed Perl interpreter (http://www.perl.org). The list of programs required by GenSeed-HMM varies according to the type of seed employed and the assembler that will be used, as well as whether mapping of recruited reads to resulting contigs is desired. For profile HMM seeds, the following packages/programs are required: *transeq* from the EMBOSS package (Rice et al., 2000), BLAST+ (Camacho et al., 2009), and HMMER v3.0 (Eddy, 2011). For the optional mapping of recruited reads against resulting contigs, Bowtie2 (Langmead and Salzberg, 2012). GenSeed-HMM requires at least one installed DNA assembler and is compatible with the following programs: SOAPdenovo (Luo et al., 2012), ABySS (Simpson et al., 2009), Velvet (Zerbino and Birney, 2008), Newbler (GS *De Novo* Assembler, Roche 454 Life Sciences, available under request at http://my454.com/contact-us/software-request.asp), and CAP3 (Huang and Madan, 1999). If Newbler is to be used, programs *sfffile* and *sffinfo* (both distributed by Roche 454 Life Sciences) and *splitter* (from EMBOSS) are also required. Progressive assemblies were performed using GenSeed-HMM. Several parameter sets were tested to optimize assembly results. Parameters used in the final experiments reported here are: -assembler newbler -ext_seed_size 30 -max_contig_length 10000 -threads 20 -clean no -mapping yes -blastn_parameters “-evalue 0.0001 -num_threads 20 -dust no -perc_identity 85” -no_qual. Specific profile HMM seeds (Supplementary File 1) were used throughout this work and specified on GenSeed-HMM with parameter –seed.

### Profile HMM construction

For profile reconstruction, all available sequences corresponding to previously reported viral proteins (VP) of *Alpavirinae* (Roux et al., 2012), named VP1, VP2, VP3, and VP4, were retrieved. Multiple sequence alignments (MSA) of each group of proteins were created using MUSCLE (Edgar, 2004) with default parameters, and the alignments were manually inspected with Jalview (Waterhouse et al., 2009) to identify conserved regions. The MSA was appended with the respective (VP1, VP2, VP3, or VP4) proteins from *Gokushovirinae* and *Pichovirinae* in order to determine whether identified conserved regions were subfamily specific. Specific regions on VP1 (Supplementary Figure 1) and VP4 (not shown) were selected and profile HMMs were built using *hmmbuild* from the HMMER package (Eddy, 2011). We adopted a nomenclature composed of the viral protein name (e.g., VP1) plus the region (e.g., R4) of the multiple sequence alignment chosen to build the respective profile HMM used as seed.

### Assembly evaluation and cross-similarity analysis of contigs reconstructed with different profile HMM seeds

Contigs assembled with GenSeed-HMM were analyzed with in-house scripts to list and calculate contig lengths and generate contig size ranks. Contigs reconstructed by progressive assembly using GenSeed-HMM with different profile HMM seeds were sorted in descending order by length and submitted to an all-vs-all *blastn* similarity search. Clusters included contigs presenting at least 90% similarity at the nucleotide level, covering at least 90% of the length of the shortest contig. Contig clusters were used to evaluate consistency between assemblies based on different profile HMM seeds to identify the potential minimum contig set.

### Taxonomic assignment of contigs

For taxonomic assignment of assembled contigs, *blastx* similarity search was used to compare assembled contigs against all reference *Microviridae* proteins (Roux et al., 2012) with a cutoff *E*-value of 1e-20. The top 10 hits were manually checked for consistency and taxonomic assignment was given to the subfamily to which all significant hits were observed. Taxonomic assignment was set to all subfamilies matched in cases where hits with similar scores were obtained to more than one subfamily. Taxonomic assignment to each cluster was done by comparing individual contig assignments within each cluster; for all clusters, we observed 100% agreement in taxonomic classification among the contigs constituting the corresponding cluster.

### Contig distribution from different human samples

Sequence reads derived from each human donor fecal sample (Reyes et al., 2010) were mapped using Bowtie2 (Langmead and Salzberg, 2012) to the assembled contigs assembled by GenSeed-HMM using the VP1R4 seed. Mapping counts were normalized by contig length and sample sequencing effort (RPKM—Reads Per Kilobase per Million mapped reads), and log transformed. The resulting matrix was used to generate a heatmap diagram.

### Sequence analysis and annotation

All assembled contigs were submitted to an automatic annotation pipeline using the development version EGene 2, derived from the EGene platform (Durham et al., 2005). The pipeline starts with a gene prediction step using Glimmer 3.02 (Delcher et al., 2007) using a training set composed of *Alpavirinae* proteins (Roux et al., 2012). All translated products were then submitted to *blastp* searches against the non-redundant (nr) database and a database composed of proteins derived from *Microviridae*. Hits were considered positive when presenting *E*-values below 1e-6. Protein domains and families were subsequently identified via InterPro (Mitchell et al., 2015) searches. In the specific case of contig annotation from the VP1R4 assembly, annotation has been manually curated to find missing and/or truncated ORFs. Automatic annotations and all stored evidence for contigs are publicly available at http://www.coccidia.icb.usp.br/alpavirinae.

### Phylogenetic analysis

For each contig assembled using the VP1R4 profile HMM seed, the complete or partial VP1 sequence was identified, translated and used for phylogenetic analyses. Two sets of analyses were done: one using only complete VP1 proteins, while the other used only a conserved region present in all assembled contigs consisting of approximately 75 amino acids having the VP1R4 region at the C-terminus. Each set of proteins was complemented with reference VP1 proteins from published datasets (Roux et al., 2012) and GenBank-deposited datasets (see Supplementary Table 1) belonging to other *Microviridae* subfamilies: *Gokushovirinae, Pichovirinae*, and genus *Microvirus*. Protein alignments were performed using MUSCLE (Edgar, 2004) and manually edited using Jalview (Waterhouse et al., 2009). Phylogenetic analyses were performed using maximum-likelihood (ML) in RAxML 8.2.0 (Stamatakis, 2006) The best-fitting amino acid substitution model for each set was obtained with ProtTest 3.4 using the AIC statistic for model selection (Darriba et al., 2011). Finally, support for nodes in ML trees was assessed by bootstrap analysis with 100 pseudoreplicates and support values were added to the master ML tree.

### Comparison of progressive vs. global assembly

To compare progressive assemblies with the global assembly counterparts, we ran Newbler as a standalone application, with default parameters, using the complete read datasets for single-end 454 (human fecal samples) and paired-end Illumina (sewage samples) data. For the latter, assembly was performed taking into account paired-end information in order to generate the best possible global assembly. All contig sequences obtained were translated into the six possible reading frames using *transeq* and then used as a dataset for *hmmsearch* (HMMER3 package) using the VP1R4 profile HMM as query. Contigs coding for HMM-positive protein sequences were identified and their nucleotide sequences used for size ranking and comparison to contigs assembled by GenSeed-HMM.

### Coverage analysis

Read alignment (SAM) files produced by GenSeed-HMM were loaded onto Tablet (Milne et al., 2013; https://ics.hutton.ac.uk/tablet/) and used to generate base-by-base coverage files for each assembled contig. Coverage information of global assembly was obtained from alignment information files produced by Newbler, and average per-base-coverage for each contig was calculated. VP1R4-containing contigs, derived from the global and progressive assembly, were pooled together and submitted to a *blastn* all-vs-all similarity search. Contigs that were at least 97% identical at the nucleotide level over at least 90% of the length of the shortest contig were clustered.

## Results

### GenSeed-HMM implementation and execution

**Figure 1:**
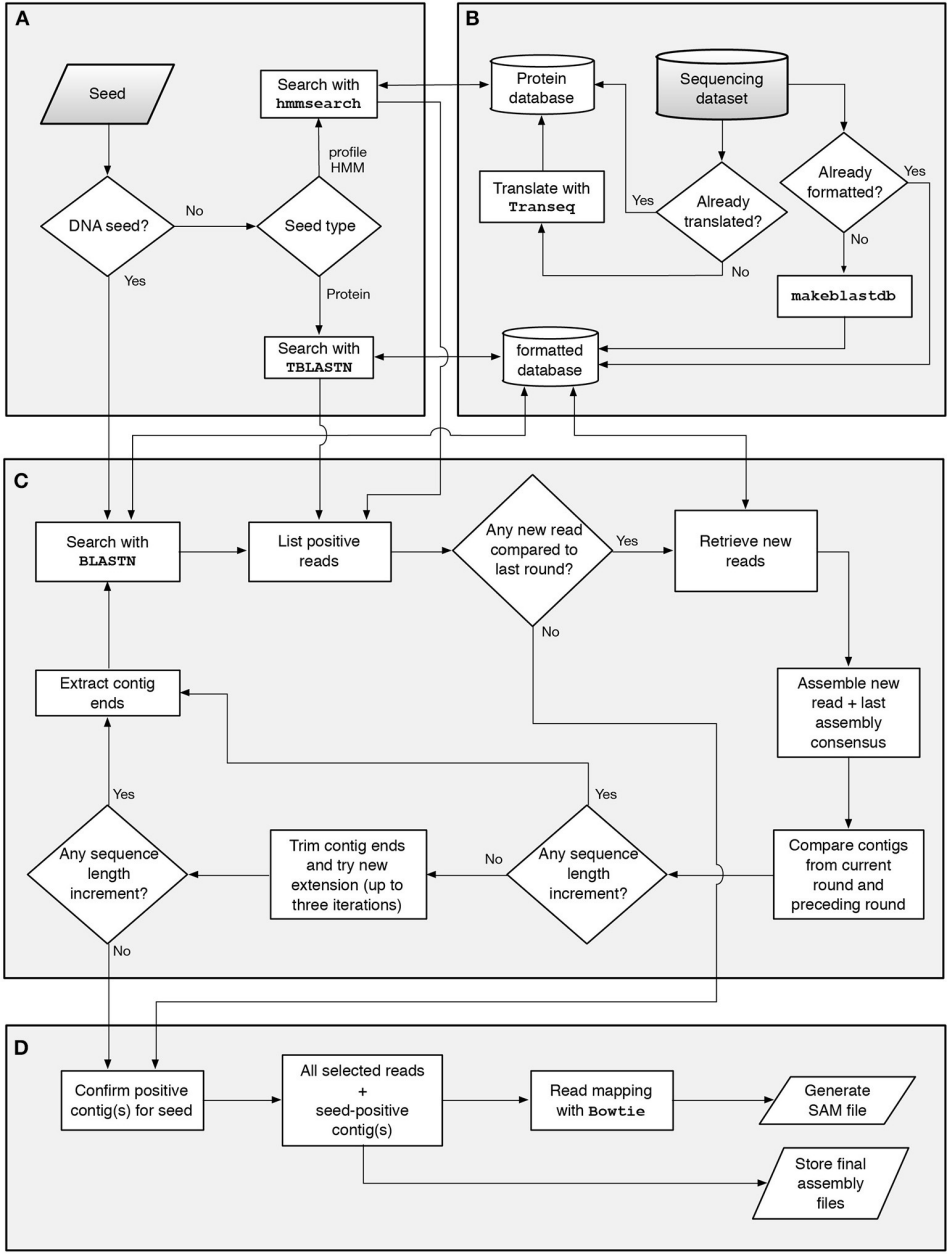
**Workflow of the seed-driven progressive assembly process**. GenSeed-HMM automatically identifies the type of starting seed **(A)**. The sequencing read database is indexed and, if needed, translated **(B)**. DNA, protein or profile HMM seeds are then used to select reads from the database using *blastn, tblastn*, or *hmmsearch*, respectively. The list of positive reads is introduced into the progressive assembly cycle **(C)**. The reads retrieved from the database are assembled and the contig ends are extracted and used as new seeds in an iterative process. The progressive assembly contains several checkpoints and is completed when a set of finishing criteria are fulfilled. In the final procedure **(D)**, all contigs are checked in regard to the presence of the starting seed and final files are stored.

GenSeed-HMM is a completely revised and extended version of the previously described GenSeed program (Sobreira and Gruber, 2008). With the advent of next-generation sequencing (NGS) platforms, the ability to use up-to-date sequencing data and DNA assemblers became an essential feature for any sequence reconstruction program. Hence, several improvements over GenSeed's original implementation have been implemented: (1) in addition to CAP3, GenSeed-HMM can now use Newbler, Velvet, SOAPdenovo, or ABySS as third-party assemblers; (2) input formats now include FASTA, FASTA.QUAL, FASTQ, and SFF, including the possibility of using quality values for CAP3 and Newbler; (3) instead of BLAST, GenSeed-HMM now uses BLAST+, a new version of the BLAST suite that uses the NCBI C++ Toolkit and presents several performance and feature improvements; and (4), in addition to DNA and protein sequences, profile HMMs can now be employed as seeds by using HMMER3, a package that performs similarity searches using profile HMMs as queries, with a performance comparable to BLAST. GenSeed-HMM automatically detects seed type (DNA, protein or profile HMM; Figure 1A). The program accepts as input a sequencing dataset generated by any of a variety of platforms and, in our experience, GenSeed-HMM can effectively reconstruct sequences using datasets originating from Sanger, 454, or Illumina technologies, with reads as short as 35-bp (data not shown). The dataset format is automatically identified and, if necessary, converted to FASTA. The database for BLAST+ is then generated by *makeblastdb* (from the BLAST+ package). If a profile HMM is used as a seed, the sequencing dataset is submitted to a six-frame translation using *transeq* (from the EMBOSS package). GenSeed-HMM performs these steps only once and reuses previously generated files in subsequent runs (Figure 1B). The progressive assembly cycle (Figure 1C) starts either with a similarity search (*blastn* for DNA seeds, *tblastn* for protein seeds) or with a profile search (*hmmsearch* for profile HMM seeds) against the translated sequencing dataset. Whatever the type of similarity search, a list of hits is obtained and used to retrieve all positive reads (and, if applicable, their quality scores) using internal sequence indexer and retriever functions. The reads are then assembled and the contig ends are used as nucleotide seeds for the subsequent assembly round. These sequences, called extension seeds, can have a variable user-defined length compared to the original seed. All assembly steps use the recruited reads combined with the contig sequence from the previous round, to guarantee that previously obtained sequences will not be disrupted by the incorporation of new reads. The use of multiple seeds is implemented in GenSeed-HMM and if two or more growing contigs overlap at a given assembly cycle, the assembler merges them into a newly generated contig. At any cycle there are checkpoints that determine if new reads have been recruited since the last round and if the resulting contigs increased in length compared to the previous round. The progressive assembly process is interrupted if any one of four conditions is satisfied: (1) the contig has reached the optional user-defined maximum length; (2) the optional user-defined number of assembly iterations has been reached; (3) no new read has been recruited by the current extension seeds compared to the preceding round; or (4) no sequence length increment has been observed since the previous round. In this latter case, GenSeed-HMM executes an iterative trimming routine, which may help overcome extension halts caused by sequencing errors. Briefly, the program iteratively trims the ends of the contig, removing an amount of bases corresponding to 25% of the extension seed length at a time (for a maximum of three steps), and tries to repeat the assembly after each trimming phase. If any step succeeds at recruiting new reads and increasing the contig length, the progressive assembly process is resumed. Conversely, the assembly process is finished and GenSeed-HMM proceeds to the final processing and file storing routines (Figure 1D). At the final checking procedure, all contigs assembled at the last round are checked for the presence of the original seed, with only seed-positive contigs being stored. Several processing files, including those generated in the intermediate assembly steps can be stored if specified by the user. Since the assembly is progressively generated, no true assembly files (e.g., those listing meaningful contig qualities, graph information, etc.) are produced. Thus, if required by the user, GenSeed-HMM invokes *bowtie2* to map all recruited reads onto the final contigs. A SAM file is then generated and stored, and can be inspected using a graphical viewer for sequence assemblies and alignments such as *tablet* (Milne et al., 2013).

### Profile HMM design and use in progressive assembly

**Figure 2:**
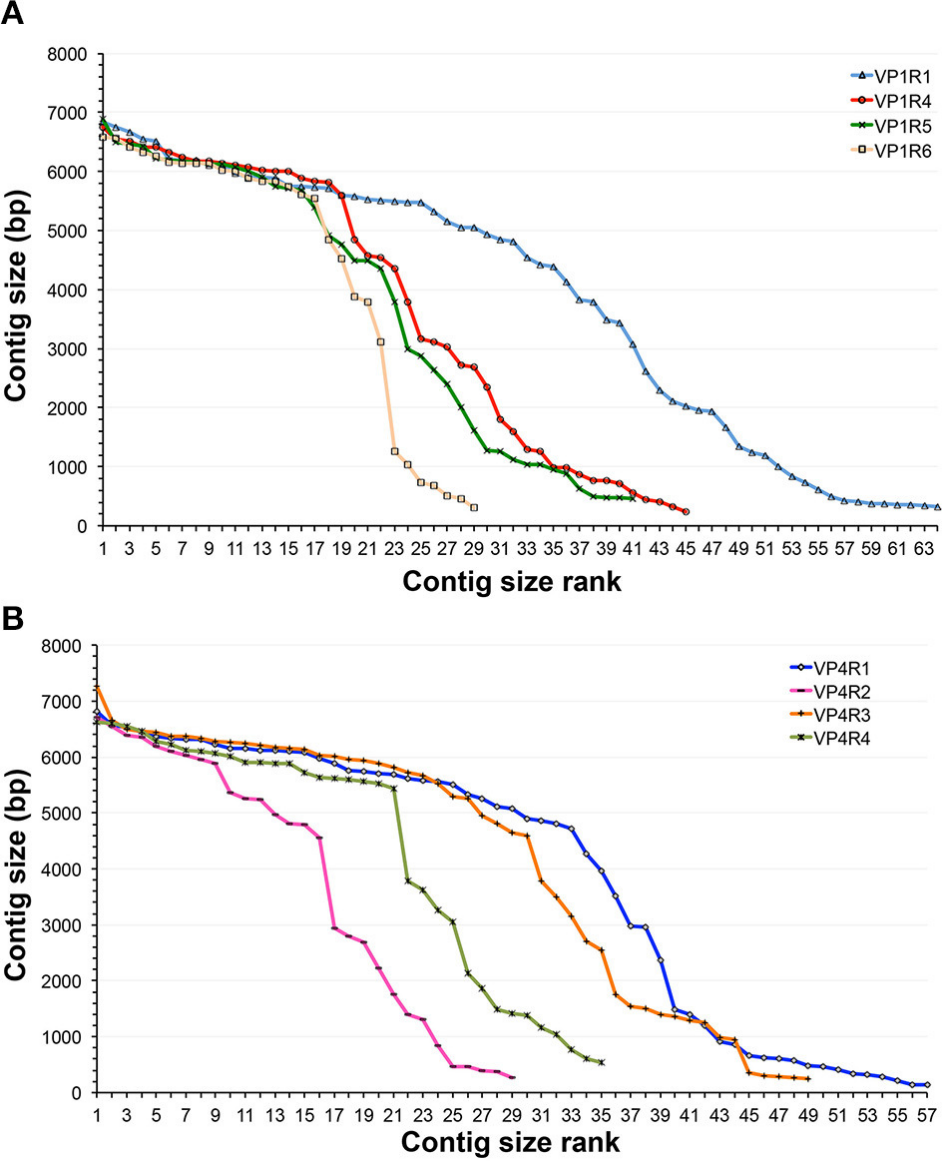
**Comparison of progressive assembly using different HMM seeds**. Contig profiles obtained by progressive assembly with GenSeed-HMM using a 454 dataset from fecal samples from human patients (Reyes et al., 2010) and profile HMM seeds derived from *Alpavirinae* major capsid protein VP1 **(A)** and replication initiation protein VP4 **(B)**. Contigs are ranked in decreasing order of size. Each marker represents a distinct contig. Profile HMMs used as seeds are depicted.

Since evolutionary processes may impose different selection pressures, proteins may evolve at different rates and even specific domains can present different degrees of conservation. We used GenSeed-HMM in order to identify potential, previously unidentified viruses belonging to the *Alpavirinae* subfamily, recently identified as part of human gut microbial communities. After analyzing the conservation of the *Microviridae* VP1, VP2, VP3, and VP4 proteins, we decided to initially use a dataset of VP1 and VP4 proteins from available *Alpavirinae* assembled genomes (Roux et al., 2012) to identify conserved regions and then, by appending homologous proteins from *Pichovirinae* and *Gokushovirinae*, select regions with specificity to the subfamily *Alpavirinae*. VP1 is the major capsid protein, a highly conserved protein that has been used as a phylogenetic marker of the group, while VP4 is a genome replication initiation protein and is more diverse in sequence than VP1 (Roux et al., 2012). A total of four distinct regions were selected for each of VP1 (Supplementary Figure 1) and VP4 (not shown) proteins. All profile HMMs were independently tested as seeds in progressive assembly assays using GenSeed-HMM and a dataset derived from viral-like particle (VLP) purification from human fecal samples (Reyes et al., 2010). This dataset is composed of approximately 1.2 million reads generated on the 454 platform, presenting a post-trimming average size of 256 bases. Initially, all contig sets were evaluated by a simple quantitative criterion, considering solely the contig size rank. In the case of VP1 (Figure 2A), the VP1R1 profile HMM showed the best performance, with the largest number of long contigs, followed by VP1R4, VP1R5, and VP1R6, respectively. For the VP4 protein (Figure 2B), VP4R1, and VP4R3 showed the best results, with VP4R4 and VP4R2 clearly resulting in a much lower number of long-sized contigs. To check the robustness of the method to different NGS technologies, the same profile HMMs were also tested as seeds with a metagenomic dataset derived from raw sewage, composed of 53.5 million Illumina paired-end reads with a post-trimming average size of 92 bases. Either with VP1 (Supplementary Figure 2A) or VP4 (Supplementary Figure 2B) profile HMM seeds, the results were very similar to those observed with fecal samples, with VP1R1, VP4R1, and VP4R3 generating longer contigs than the other seeds.

**Figure 3:**
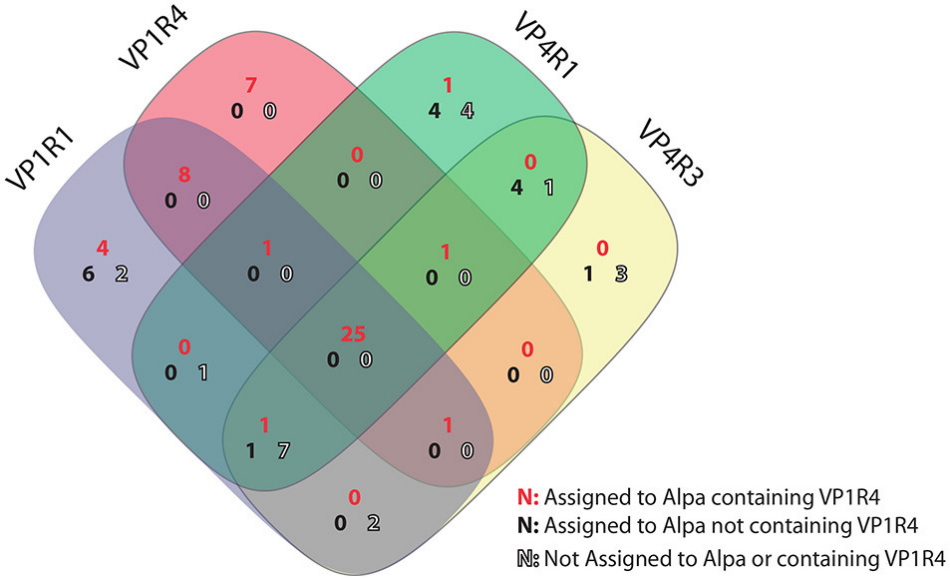
**Consistency among HMM seeds**. Venn diagram representing shared contigs reconstructed by progressive assembly using GenSeed-HMM with profile HMM seeds VP1R1, VP1R4, VP4R1, and VP4R3. Contigs were included in the same cluster when presenting at least 90% similarity at the nucleotide level covering at least 90% of the length of the shortest contig. Contigs were then taxonomically classified by *blastx* to reference proteins from *Microviridae* and searched for the presence of the VP1R4 seed using *hmmsearch*. A large percent of shared contigs among all four seeds is observed and belonging to *Alpavirinae* genomes covering the VP1R4 seed. Notice that contigs that were not present within the VP1R4 seed were usually not assigned to *Alpavirinae* (low precision) or do not contain the VP1R4 region, suggesting potential shorter non-overlapping contigs.

The variability in the sequence reconstruction ability by the different HMM seeds led us to investigate how the different assemblies compared to each other in terms of their contig sequences. Thus, we used the top four performers (VP1R1, VP1R4, VP4R1, and VP4R3) to run *blastn* all-vs-all similarity searches followed by sequence clustering. It is noteworthy that despite the overall contig size rank variation (Figures 2A,B), from the total of 85 de-replicated contigs, 25 were identified as being assembled independently by all four assemblies, whereas using either VP1 or VP4 seeds showed the second highest overlap (Figure 3). Therefore, highlighting that regardless of the differences observed in contig size rankings, the assemblies were highly consistent, even though they were derived from profile HMMs built from distinct regions and/or proteins. The only other overlap with a significant number of contigs involved nine contigs shared between VP1R1, VP4R3, and VP4R1. However, further taxonomic assignment (see section below) showed that only two of these contigs were assigned to *Alpavirinae*, suggesting lower precision for these seeds.

A further analysis of assembly performance, in particular regarding to VP1R4 seed, showed that some contigs covering this region have not been assembled using the corresponding seed, but rather by one or more other seeds (Figure 3). For instance, 14 contigs were assembled exclusively by the VP1R1 seed, with 10 of them being assigned to the *Alpavirinae* subfamily, and four of those covering the VP1R4 region. A detailed analysis of these latter contigs confirmed that the VP1 proteins of this subset were too divergent to be detected by the VP1R4 seed. This phenomenon was observed in all contigs assigned to *Alpavirinae*, but not assembled by the VP1R4 seed (Figure 3—represented by black numbers). Another interesting observation was the fact that six contigs assigned to *Alpavirinae*, and containing the VP1R4 seed, have not been assembled when using this particular seed (Figure 3—represented by red numbers). In this case, we identified three events where the VP1R4 seed successfully detected the corresponding reads, but due to a very low coverage on this specific region, Newbler was unable to generate an assembled contig in the first assembly cycle. Finally, for the remaining three events, we identified short sequences on the VP1R4 set of contigs that were very similar (but slightly below our 90% threshold) to contigs assembled by the other seeds. By comparing the read coverage of the shorter contigs with their longer counterparts, it became clear that the ends of the shorter contigs presented lower coverage than the corresponding regions in the longer ones, indicating premature extension stoppage events (data not shown). This seems to be a consequence of the directionality of the progressive assembly method. The assembler is able to extend the growing sequence in one direction, but, due to base discrepancies biased at a particular end of one or more reads, the resulting alignment graph ends up containing a so-called bubble, precluding the assembler from extending the sequence in the opposite direction. By precisely identifying the few different assembly failures, we expect to develop new routines that could automatically handle these problems, should they happen, during an execution.

### Taxonomic assignment of assembled sequences

Given that the aim was to reconstruct *Alpavirinae* genomes from metagenomic datasets, we wanted to address the sensitivity and precision of the methodology. The sensitivity (number of *Alpavirinae* associated contigs from the total number of *Alpavirinae* viruses in a given dataset) and precision (number of *Alpavirinae* associated contigs from the total number of contigs assembled with a given seed) will be dependent on the specific profile HMM seed used, the quality and coverage of the sequencing and the specific parameters used. To address this point we used similarity searches with *blastx* against a reference dataset of *Microviridae* proteins (Roux et al., 2012), together with sequence clustering analysis, and we were able to classify the contigs into three subfamilies of *Microviridae* (Table 1). Since all HMMs have been originally built toward *Alpavirinae*-conserved regions, a predominance of sequence assignment to this subfamily was expected. In fact, this was the most prevalent taxon of the reconstructed sequences for all seeds. However, with the exception of VP1R4, which presented 100% precision, the three remainder HMMs also led to assembled *Gokushovirinae* and *Pichovirinae* sequences, with precision values varying from 72.3 to 79.7% (Table 1). The unambiguous taxonomic assignment of VP1R4-derived contigs was confirmed by phylogenetic analysis (see below). Clustering analysis showed that among the four assemblies it was possible to generate a total of 85 non-redundant non-overlapping contigs (Table 1). However, this result does not necessarily imply that there is a total of 85 different originating viral entities in the sample, since each assembly resulted in a number of partial, shorter contigs centered on the specific profile HMM seed that could be generated from the same virotype but, due to sequencing coverage or other factors affecting assembly, were not extended enough to identify overlaps with contigs produced by other profile HMMs.

**Table 1:** **Taxonomic assignment of contigs (human fecal data, progressive assembly) and classification precision and sensitivity**.

**Subfamily**	**Profile HMM seed**				
	**VP1R1**	**VP1R4**	**VP4R1**	**VP4R3**	**Total**^a^
*Alpavirinae*	47	43	38	34	65
*Gokushovirinae*	11	0	11	10	17
*Pichovirinae*	1	0	1	1	1
Gokush*/*Alpa^b^	0	0	1	2	2
Total	59	43	51	47	85
VP1R4-positive	40	43	23	24	49
Sensitivity for *Alpavirinae*	(47/65) 72.31%	(43/65) 66.15%	(38/65) 58.46%	(34/65) 52.31%	
Precision for *Alpavirinae*	(47/59) 79.66%	(43/43) 100.00%	(38/51) 74.51%	(34/47) 72.34%	
Sensitivity for VP1R4	(40/49) 81.63%	(43/49) 87.76%	(23/49) 46.94%	(24/49) 48.98%	
Precision for VP1R4	(40/59) 67.80%	(43/43) 100.00%	(23/51) 45.10%	(24/47) 51.06%	

*Contigs generated by GenSeed-HMM with the respective profile HMM (VP1R1, VP1R4, VP4R1, and VP4R3) were compared against all reference Microviridae proteins (Roux et al., 2012) using blastx with a cutoff E-value of 1e-20. When hits with similar scores were obtained to more than one subfamily, taxonomic assignment was set to two subfamilies. Contigs were also evaluated for the presence of VP1R4 region by hmmsearch, and the number of positive contigs is shown*.

a *Total number of De-replicated contigs (See Figure 3) that belonged to a given taxonomic assignment*.

b *Represents a set of two contigs where the best BLAST hit annotation was below the E-value cutoff and they were equally distant by percent identity to Gokushovirinae and Alpavirinae, so no single assignment was possible*.

Assessing the sensitivity of the different seeds constitutes a challenge since it is impossible to address the real total number of expected *Alpavirinae* genomes. In order to have an approximation to this value we analyzed two different metrics that should constitute an approximate range of the actual sensitivity. As an upper bound, we used the number of total contigs (independently of the seed used) assigned to *Alpavirinae* (*n* = 65; Table 1), which is very likely to give an over-estimated sensitivity value due to independent contigs formed by different seeds that originate from a single viral entity, as mentioned above. The lower bound was done specifically for the VP1R4 seed and consists of the number of contigs from all assemblies that covered the region used to build the VP1R4 HMM (*n* = 49; Table 1). By the estimation of these contig numbers it was possible to calculate that the sensitivity for the VP1R4-based assembly should be between 66.2 and 87.8% (Table 1 and Figure 3). In a similar way, we calculated the sensitivity and precision of the progressive assembly performed on the sewage data (Supplementary Table 2) in this case we observed for the VP1R4 seed a similar precision (99.67%) and a sensitivity between (35.8–91.6%), the wider range is due to the higher number of total contigs (2480) due to the larger dataset with shorter reads generating a more fragmented assembly.

The advantage of using profile HMMs as seeds for progressive assembly is clear when the same data is investigated by protein similarity searches. With that aim, each of the 33 full-length VP1 sequences from Roux et al. (2012) was compared by *blastp* similarity searches (Supplementary Table 3) to our 45 complete or partial VP1 sequences originated from the VP1R4 assembly. Even using an *E*-value of 1e-6, which is not particularly stringent, we have found that each of the 33 proteins matched only 16–37 of the 45 novel sequences. This shows that a single profile HMM seed derived from a short VP1 region was much more sensitive than any of the 33 complete protein sequences for the detection of novel *Alpavirinae* sequences. Because these full-length sequences include stretches conserved across proteins from other viral subfamilies, they would probably yield a lower precision. To establish a fair comparison between protein and profile HMM seeds, we assessed the detection ability of GenSeed-HMM using sequences restricted to the VP1R4 seed region (coordinates 799–816—see Supplementary Table 3). The observed individual detection rate was much lower indeed, varying from 0 to 4 sequences with a cutoff of 1e-6, and 0–15 with a cutoff of 1e-2. Although these tests were performed using *blastp* directly instead of running GenSeed-HMM, they show, in a specific manner, that the nature of the seed is what is leading to a difference in sensitivity. These results indicate that a seed-driven assembly based on a single protein sequence is limited to the information contained on that sequence itself, while profile HMMs, by incorporating the variability of a full set or family of sequences, present higher sensitivity and wider range of detection.

### Using multiple profile HMM seeds

As presented above, no single profile HMM seed was able to assess the true viral complexity of the sample (Table 1 and Figure 3). Since GenSeed-HMM can use multiple seeds in a single execution, we decided to run a preliminary comparative analysis to evaluate the ability of single and multiple profile HMMs to reconstruct viral genomes. The profile HMMs were employed either individually or in combination of two or four seeds to progressively assemble sequences from the 454 dataset from human fecal samples (Reyes et al., 2010). All identified *Alpavirinae*-specific contigs were submitted to contig size rankings (Supplementary Figure 3), with VP1R1 exhibiting the best overall contig size profile, in agreement with what had been previously observed without filtering out contigs belonging to other *Microviridae* subfamilies (Figure 2). When using the VP1R4 and VP4R1 seeds, derived from two distinct viral proteins, the obtained profile was clearly better than the profiles observed with the use of any of the individual seeds. The use of pairs of seeds derived from the same protein (e.g., VP1R1/VP1R4 or VP4R1/VP4R3) did not show relevant improvement over individual seeds (data not shown). Nevertheless, it is noteworthy that a combination of the four seeds yielded the best assembly. This result strongly suggests that a rational combination of profile HMM seeds can be used to unravel the true viral diversity in a sample. Is important to highlight that the number of close to full-length contigs (around 6 kb) does not change with multiple seeds, suggesting that the longest contigs were recovered with either one or multiple seeds.

### Phylogenetic analysis

**Figure 4:**
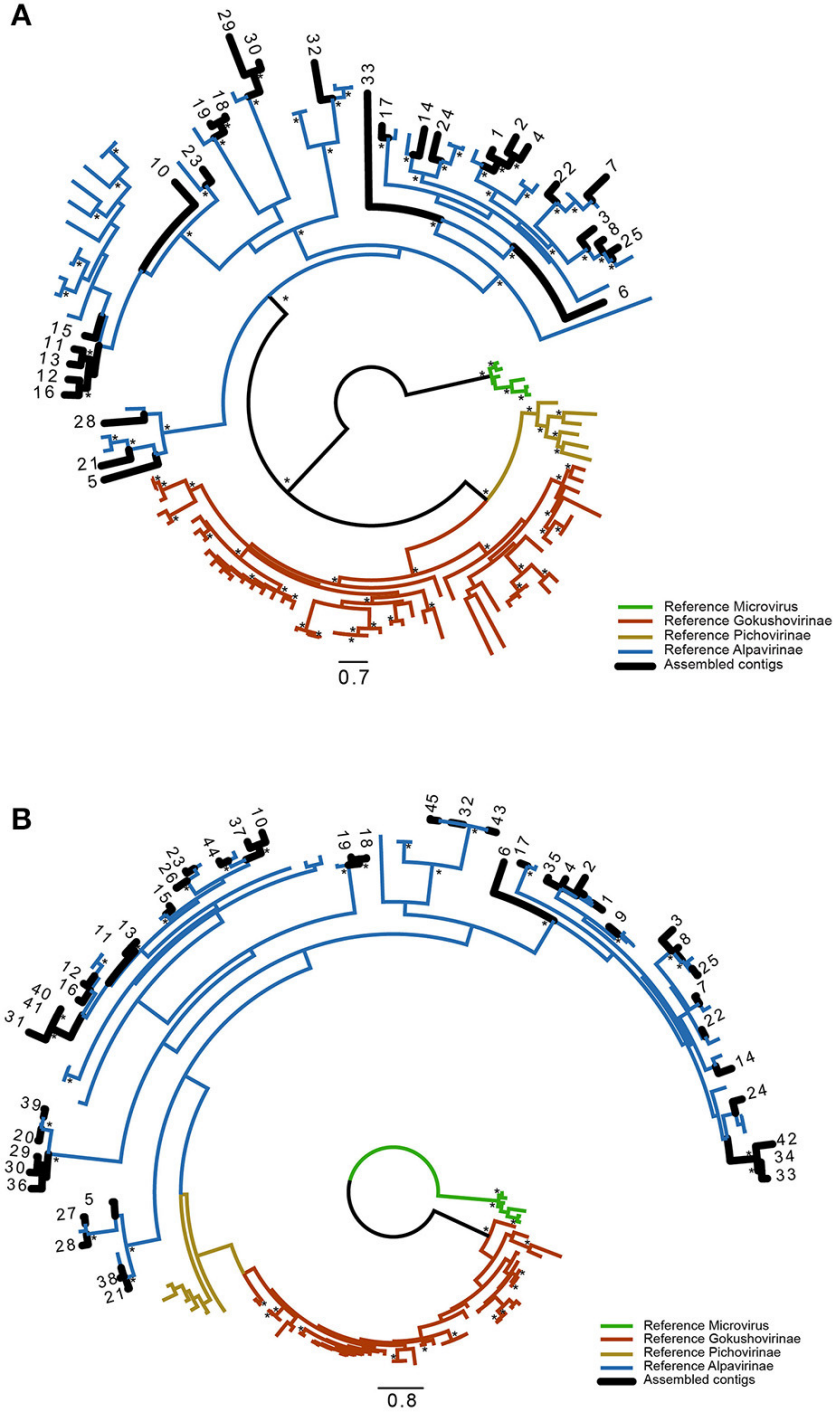
**Phylogenetic analysis**. Maximum likelihood phylogenetic analysis of **(A)** full-length VP1 protein and **(B)** a shorter region comprising only the VP1R4 HMM region. Sequences were translated from the contigs reconstructed by GenSeed-HMM using the VP1R4 seed. Different subfamilies of the *Microviridae* family are depicted in distinct colors, references were obtained from Roux et al. (2012). Branches represented by sequences derived in this work are labeled in black. Asterisks in the nodes indicate bootstrap values higher than 70%. Numbers represent contig numbers as observed in Figure 5.

Using an automated processing pipeline, all sequences assembled with the four profile HMMs were annotated. This automatic annotation was the basis for the identification of the VP1 genes and the respective translation to the corresponding protein sequences. Since we have determined that only the VP1R4 assembly generated sequences restricted to the *Alpavirinae* subfamily (see previous section), this particular annotation set was manually curated and used to produce a dataset of complete and partial VP1 protein sequences. For phylogenetic inference, we used a reference dataset of *Microviridae* proteins (Roux et al., 2012) and sequences publicly available on GenBank (Supplementary Table 1). Since some of the assembled contigs represented incomplete genomes and covered slightly more than the VP1R4 region, we performed two phylogenetic reconstructions using either full-length VP1 proteins or sequences covering approximately 75 amino acids with the VP1R4 region at the C-terminus. The tree containing 28 novel full-length sequences (Figure 4A) showed better bootstrap support than that for a tree inferred with 45 shorter sequences (Figure 4B), but both converged to the same topology. Both trees clearly separate the different subfamilies and show that all assembled contigs are completely specific to the *Alpavirinae* subfamily, thus corroborating our previous similarity-based taxonomic analysis. In addition, these novel sequences were not confined to a few clades, but rather spread in almost all clades containing reference sequences described by Roux et al. (2012) and/or available on GenBank suggesting that this subfamily is highly diverse and broadly dispersed in humans.

### Intra- and inter-personal distribution of novel *Alpavirinae* sequences

**Figure 5:**
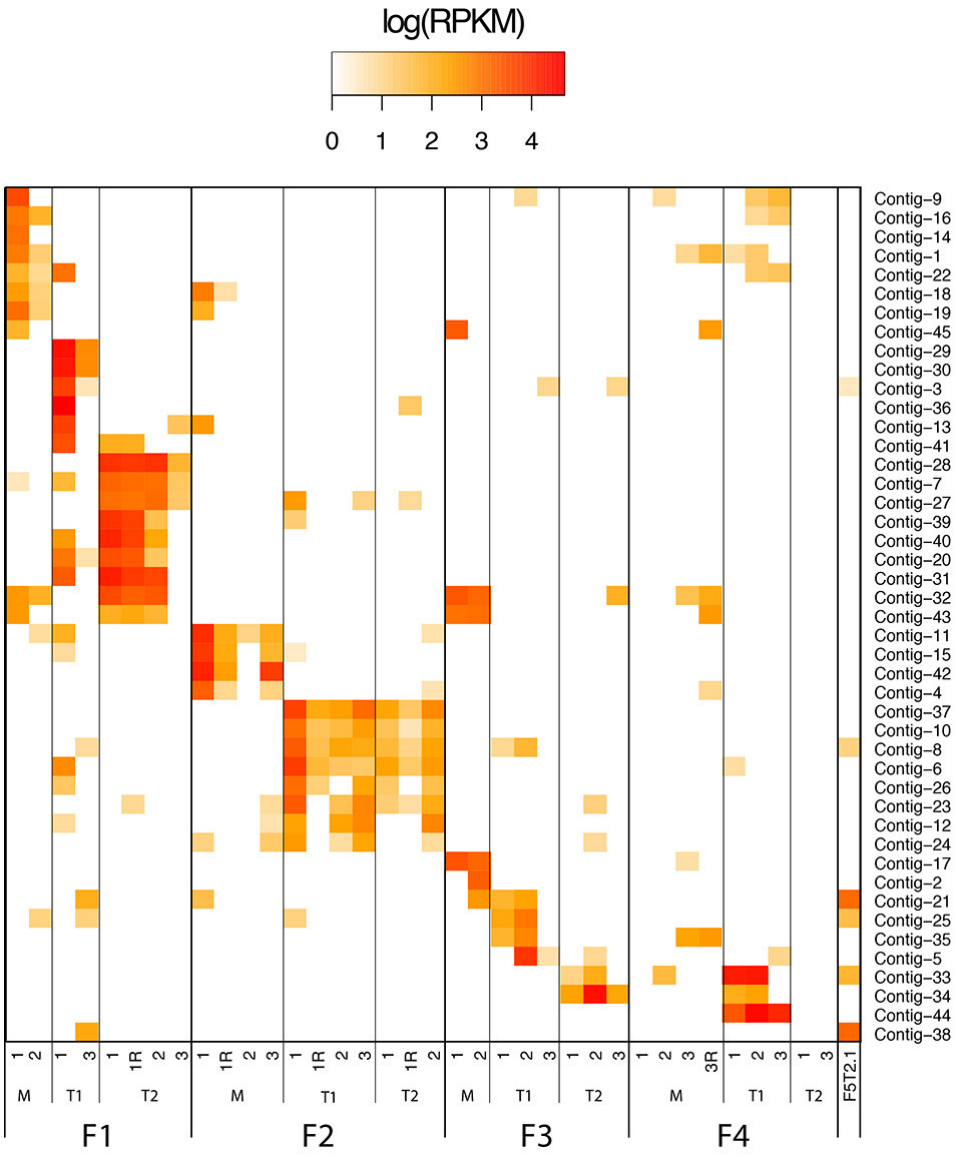
**Read abundance of contigs**. Heatmap diagram representing read abundance in contigs reconstructed by progressive assembly with GenSeed-HMM and the VP1R4 seed. Fecal biospecimens were collected from different families (F1–F4) composed of monozygotic twins (T1 and T2) and their respective mothers (M). Time points of sample collection and technical replicates (R) are depicted. Data source: 454 dataset from fecal samples of human patients (Reyes et al., 2010).

Read abundance in contigs reconstructed by progressive assembly with GenSeed-HMM using the VP1R4 seed was used to assess the distribution of the newly characterized *Alpavirinae* sequences across the different human donor fecal samples. The novel *Alpavirinae* sequences showed highly conserved intrapersonal patterns along different time points (Figure 5), similarly to what has been previously observed for whole viromes (Reyes et al., 2010). Conversely, interpersonal viral variations were much higher, with few cases of shared contigs even between twins of the same family, except for the twins on family 2. It has been suggested that the *Alpavirinae* subfamily is linked to genera of the Bacteroidetes phylum (Krupovic and Forterre, 2011). Our results show that distinct individuals harbor different amounts of each of these viruses (Figure 5), which are usually not closely phylogenetically related (Figure 4B), suggesting that they are probably associated with different Bacteroidetes taxa.

### Progressive vs. global assembly

**Figure 6:**
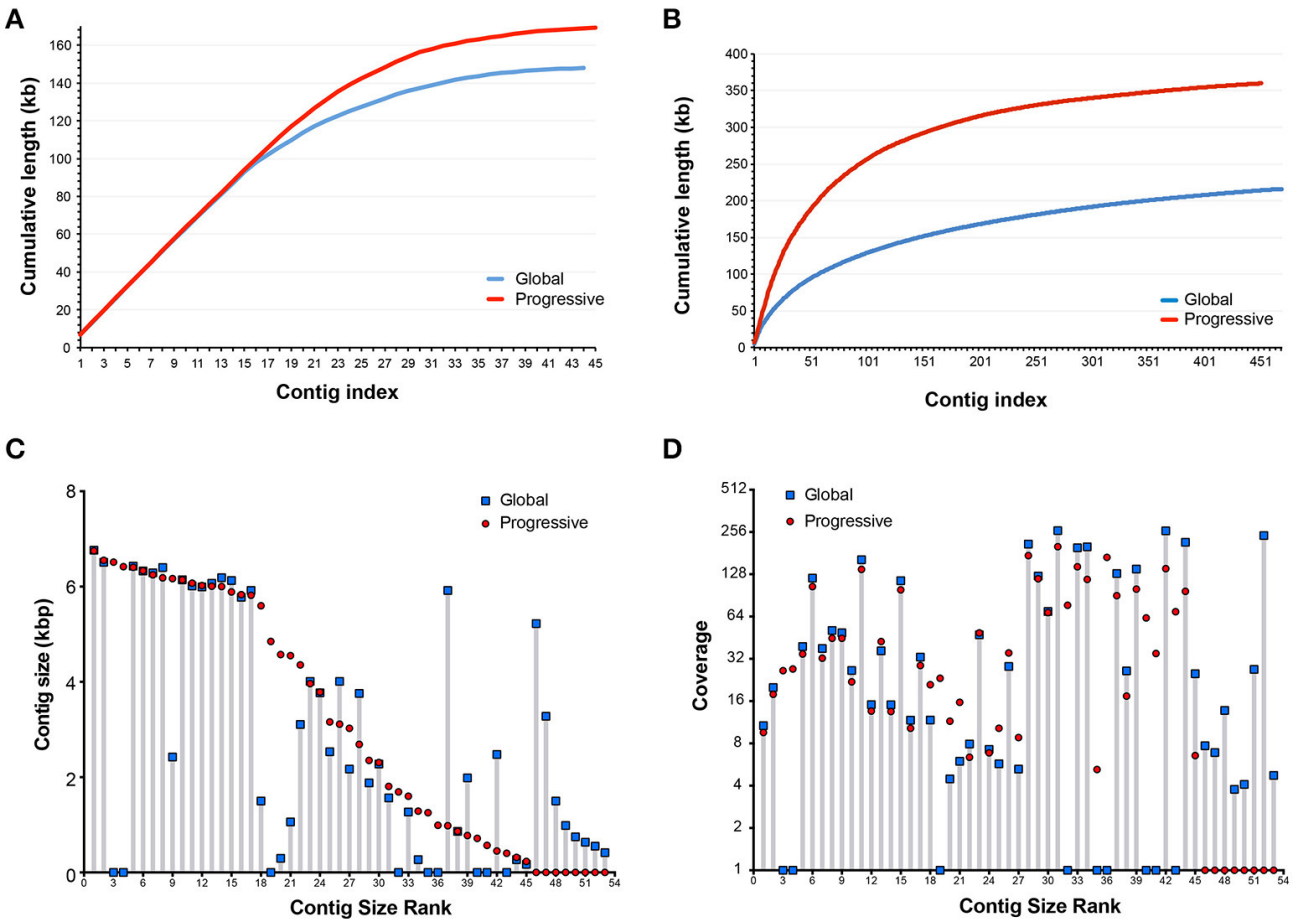
**Comparison between global and progressive assembly**. Comparison of cumulative contig lengths using progressive assembly with GenSeed-HMM and VP1R4 HMM seed and global assembly with Newbler. Data sources: **(A,C,D)** 454 dataset from fecal samples of human patients (Reyes et al., 2012); **(B)** Illumina dataset from a sewage treatment plant at the municipality of Taboão da Serra, São Paulo, Brazil (unpublished data). Contigs from progressive assembly and VP1R4-positive contigs from global assembly were clustered at 97% identity over at least 90% of the shortest contig, each cluster consisted at most of one contig from each dataset. A total of 53 clusters were generated, nine unique for the progressive assembly and eight unique for global assembly. Plotted is the comparison in lengths **(C)** and coverage **(D)** for related contigs obtained by progressive and global assemblies and ranked by size.

To address how progressive assembly performs against conventional global assembly, we compared our contigs generated using GenSeed-HMM with the VP1R4 seed to the results obtained using Newbler in a standalone execution for the same original dataset (global assembly). By selecting only contigs coding for VP1R4-positive proteins (using *hmmsearch*), a fair comparison between both assembly methods could be established. An initial analysis, based on cumulative contig lengths (Figure 6A) showed a very similar assembly performance for the 15 longest contigs obtained using the human fecal samples. From this result it can be appreciated that the progressive method clearly had a better assembly performance, characterized by a higher number of assembled bases (169 kb) than the global assembly (148 kb). The total number of contigs was similar for both approaches, with 45 contigs in the case of progressive assembly and 44 with global assembly. When the same test was applied to the Illumina dataset derived from a raw sewage sample (Figure 6B), we observed even more pronounced differences. In this case, we obtained a total of 360 kb of assembled sequence comprising 453 contigs, whereas the conventional method showed a more fragmented assembly, with a total of 216 kb and 471 contigs (See Supplementary File 2). Given the environmental nature of the raw sewage sample, a much wider viral diversity should be expected. In fact, the total number of contigs was much higher than that observed in human fecal samples. To compare the sensitivity and precision obtained with the GenSeed-HMM method and the global assembly, we performed the same taxonomic annotation and clustering analysis and clustering on these contigs. The results (Supplementary Tables 4, 5) showed equivalent numbers of sensitivity and precision with the VP1R4 seed, confirming that both strategies have similar ability to recover the viral genomes (both are based on the same assembler) but GenSeed-HMM recovers longer contigs with more efficient use of computational resources and completely centered on the target sequences.

To further characterize the consistency among the results obtained with the different strategies, we used the assembly from fecal samples for a similarity clustering at 97% identity to identify cases where the same contig was found in both assemblies. In this case, we observed that each cluster contains at most one contig from each assembly strategy. In the case of the human fecal samples, we identified a total of 53 non-redundant contigs where nine of those were unique to the progressive assembly and eight were unique to the global assembly. When comparing the contig lengths for each pair of clustered contigs (Figure 6C) it was possible to see that in 20 cases both assemblies yielded contigs of essentially the same length, while in 11 cases progressive contigs were longer than the global ones, and in five cases the opposite was observed. These findings confirmed once more that the progressive strategy was mostly capable of generating longer contigs from the same original seed than a global strategy. When comparing contig read coverage (Figure 6D) in the same dataset, it was clear that both strategies assemble contigs with similar coverage, suggesting that there is no coverage bias for the contigs assembled with the iterative progressive assembly.

## Discussion

In this work, we describe the development of GenSeed-HMM, a program that implements a seed-driven progressive assembly approach using profile HMMs as seeds, in addition to nucleotide and protein sequences. We also demonstrate the application of the implemented method for viral discovery using *Alpavirinae* as a case study. Using a previously published dataset it was possible to assemble a total of 85 *Microviridae* associated contigs, with 25 of those likely representing full viral genomes. Phylogenetic analysis showed that those novel assembled contigs contained representatives of all known clades of the *Alpavirinae* subfamily, significantly contributing to the knowledge regarding those viruses in the human gut. The use of GenSeed-HMM to assemble *de novo* viral genomes present in metagenomes provides a very important resource for the characterization and understanding of the role of different viruses and viral families in the microbial ecology of complex environments.

The current study also shows that our progressive assembly strategy generates an overall higher number of longer contigs, with read coverage equivalent to that observed in the corresponding global assemblies. This improvement could be due to effects of repetitive regions that can create chimeric contigs or even hamper global assembly, especially if these regions are longer than the average read length. Another potential problem is represented by polymorphic sequences, a feature commonly found in viral populations. In the case of global assembly, reads are analyzed all at once to construct the assembly graphs. Conversely, progressive assembly is driven by a single seed or, in the worst case, a relatively small number of seeds. This means that the search space is dramatically reduced since a very strict subset of reads is selected from the main dataset. Each assembled contig then originates two extension seeds, one from each of the contig's ends, which in turn will be used to select new small subsets of reads. Thus, each assembly round employs these relatively few reads plus the previously generated contig, which acts as a guide for sequence growth. Hence, when a repetitive region already present in a previously assembled part of the contig is reached, no newly recruited reads will disrupt the sequence already assembled. The whole process is therefore highly directional, starting from the seed sequence up to the final optimal assembly. This particular *modus operandi* is important to prevent repetitive sequences from leading to chimeric assemblies, which could entrap the process by artificially joining physically unrelated sequences.

A classical protocol for detecting viral sequences from metagenomic data is to assemble the sequence reads and then submit the contigs to BLAST searches against databases of known viral genomes or protein sequences (Cantalupo et al., 2011; Bibby and Peccia, 2013; Norman et al., 2015). This approach is severely limited by the fact that pairwise sequence comparison methods fail to detect distant evolutionary relationships, with sequence identities of around 30% seeming to represent a threshold value for identifying true homologs (Brenner et al., 1998). In the case of viral discovery, this scenario is even more challenging because of the typically high substitution rates, especially in RNA viruses that replicate through error-prone RNA-dependent RNA polymerases (RdRP). Also, the bias of sequence data available for the different viral families limits the effectiveness of similarity searches. Search methods relying on profiles are more sensitive than pairwise alignments because they incorporate broader position-specific information as well as a quantification of the range of substitutions observed across different members of the group. From several methods available, profile HMMs seem to be the most effective to detect distantly related organisms (Park et al., 1998). More recently, Skewes-Cox et al. (2014) reported a method to generate viral profile HMMs (vFams) for the detection of viruses from metagenomic data and the public release of a database composed of more than 4000 such profiles (vFam—http://derisilab.ucsf.edu/software/vFam/). These profile HMMs, constructed from MSAs covering the entire sequence of the respective proteins, showed a higher precision than BLAST searches in real metagenomic datasets, especially for more divergent viral sequences. According to the authors, vFams could be used to nucleate metagenomic assemblies with selected reads to produce longer sequences, in an approach similar to the one previously proposed by our group (Sobreira and Gruber, 2008). Another important aspect pointed out by Skewes-Cox et al. (2014) is the fact that both BLAST and HMM-based methods rely on some degree of similarity to already known viruses, meaning that updating sequence databases in a regular basis is essential for the future effectiveness of such methods, and that bioinformatics approaches based on *de novo* metagenomic assembly and *ab initio* structural prediction algorithms will have increasing importance. In this direction, there is also room to improve seed development with the possible addition of protein structure information in profile HMM design for probing deep phylogenetic associations (Deng and Cheng, 2014).

Compared to the original GenSeed program (Sobreira and Gruber, 2008), the concept of using seeds to drive the assembly process has been extended in GenSeed-HMM by the development of specific routines to deal with profile HMMs. In fact, the originally proposed nucleotide and protein seeds could drive the assembly of sequences derived from the same species or from evolutionarily close organisms. A few previous attempts using our original concepts of seed-driven and/or progressive assembly have been described, but were limited in application to fewer genomic assembly programs, DNA sequence seeds, or non-metagenomic input data (Smits et al., 2015). The original GenSeed program already used both DNA or protein sequences as seeds for iterative assembly, and GenSeed-HMM greatly expands on these capabilities by allowing the use of read data from different sequencing technologies, multiple assemblers, and profile HMM seeds. Tools such as PRICE (Ruby et al., 2013), which also use GenSeed's original assembly principles, are based exclusively on DNA seeds limiting their potential for viral discovery. Indeed, even using protein sequences, which are much more conserved than DNA, the profile HMM seed derived from a short VP1 region (VP1R4) was much more sensitive than any of the 33 complete VP1 protein sequences from Roux et al. (2012) for the detection of novel *Alpavirinae* sequences. Profile HMMs increase the spectrum of detectable organisms since they are built from MSAs derived from many organisms, encompassing a large range of variability within a single probabilistic model. The use of profile HMMs in a targeted gene assembly tool has been recently implemented on the SAT-Assembler program (Zhang et al., 2014). Using a concept similar to the seed-driven assembly described by our group and implemented in GenSeed (Sobreira and Gruber, 2008), SAT-Assembler uses the seeds to select reads from datasets and then proceeds to construct its own overlap graph for the assembly, also avoiding an all-against-all sequence comparison. However, SAT-Assembler can only generate a consensus sequence that is limited to these reads. Conversely, by means of the progressive assembly method, GenSeed-HMM can extend the sequence reconstruction as much as possible, according to user requirements. This is especially important, since the assembly is not restricted to the gene itself, but also to its flanking regions, providing genomic context information. In fact, by using the appropriate number of assembly cycles, an entire viral (or other episomes, such as mitochondrial) genome can be reconstructed using a single seed, provided that sufficient read coverage is available in the sequencing dataset, as shown in the current study.

When applied to viral discovery, simultaneous use of multiple seeds can substantially increase the sensitivity of the method by generating several starting points for assembly. If maximum sensitivity is required, combining seeds is important, as our results show that no single profile HMM seed can assess the true viral diversity present on any sequencing dataset. However, the proper choice of seeds is essential, since closely placed seeds may be inefficient for two reasons: (1) if the seeds are directed toward physically close regions, chances are that low read coverage may apply to all of them; and (2) because of the physical proximity of the seeds, specific reads recruited by a seed could overlap reads selected by other seeds, implying that the progressive assembly might give rise to something approaching a classical global assembly. Our results show that using seeds derived from different proteins is a more sensible approach. However, it is worth mentioning that using multiple seeds to attain maximum sensitivity may come at the price of lowering precision. A general recommendation for seed design includes avoiding low-complexity regions, as they would result in non-specific reads being recruited and assembled, with a consequent lack of specificity. A good compromise between sequence conservation/divergence of the region selected for profile HMM building may vary from case to case and there is no *a priori* set of rules. Delimiting the range of targeted taxa may help to define whether the profile HMM seeds should be built from selected regions or from a full-length protein sequence. Specific routines could also be implemented in future versions of GenSeed-HMM to identify and discard spurious non-specific sequences. The development of multiple seeds could also profit from a nested, hierarchical-based rationale for seed design and use that should entail aspects of viral taxonomy. For example, one could progressively use sets of seeds, initiating by using replicases, which would then lead to an informed choice of helicase and capsid-derived seeds, and so on. This would drive new virus discovery from core functions, such as replicases and capsid genes (that define viral families) to more contextual functions, such as receptor glycoproteins that would be more informative at the genus level (de Andrade Zanotto and Krakauer, 2008; Krakauer and Zanotto, 2008). We foresee that a rational protocol of profile HMM construction can be established focusing on the development of narrow- and wide-range taxonomic associations. For instance, specific profile HMMs could be built for the detection of well-delimited taxonomic groups such as subfamilies or families.

A paradigm of diagnosis, using either serological or nucleic acid-based methods, is that one can only diagnose organisms that are already known. For instance, given a pathogen to be identified by a serological assay, it is mandatory to first establish which antigens or antibodies will be the targets of detection. Likewise, PCR-based assays rely on previous knowledge of the target sequences to be amplified, and microarray-based assays, such as the Virochip, are based on known hybridization targets. Viruses are biological entities in which evolution can be observed in comparably short spans of time, given their fast rates of mutation and substitution. In fact, since the nineteen-seventies, we have witnessed the emergence of many novel human and animal diseases, such as Acquired Immune Deficiency Syndrome (AIDS) caused by the human immunodeficiency virus (HIV), Ebola virus disease (EVD), among others (Palacios et al., 2008; Wang, 2011; Rosenberg, 2015). Metagenomic data has contributed to surveys of viral diversity (Bibby and Peccia, 2013) and the discovery of novel animal (Belák et al., 2013) and human (Tang and Chiu, 2010; Siebrasse et al., 2012; Phan et al., 2015; Reyes et al., 2015) viruses. The pace of viral discovery is increasing, including many emergent zoonotic viruses pathogenic to humans (Wang, 2011; Rosenberg, 2015). Given the ever-growing amount of sequence data, the challenge is how to diagnose new potentially emerging pathogens without knowing what one is looking for. Considering that emerging viruses moved into humans from pre-existing lineages from the zoonotic pool, some key structures are conserved in essential functions such as replication and capsid proteins. HMMs able to potentially detect a wider range of taxa could be used for epidemiological surveillance, in order to monitor the emergence of new variants of already known viruses or even detect the arising of novel viruses. Profile HMMs have a series of advantages that make them ideally suited to detect sequences that have not been sampled in the original MSA within a reasonable margin of divergence, detecting related members to those used for the construction profile that likely share the same selective pressures. This feature opens up a new possibility, namely the diagnosis of novel viruses potentially pathogenic to humans and animals, without previous specific information, an approach that we refer to as *de novo* diagnosis. We believe that *de novo* diagnosis using rationally designed profile HMMs may assume a fundamental importance for epidemiological surveillance in some sentinel sites such as hospitals, sewage treatment stations, animal production facilities, and migratory bird colonies, among others. By detecting emerging viruses on these sites, it would be possible to undertake containment measures to prevent the spread of potentially devastating diseases. GenSeed-HMM provides a fast and simple implementation to run progressive assembly pipelines using profile HMMs covering the most relevant groups of viral pathogens. By combining rational design of profile HMMs and multiple GenSeed-HMM runs, one can foresee a replacement of the paradigm of conventional diagnosis.

In this work we exemplified how GenSeed-HMM could be used for viral discovery. Nonetheless, the spectrum of potential applications of the seed-driven progressive assembly method using profile HMMs is much wider. Besides viral genomes, the method is well fitted for surveys of extra-chromosomal elements such as plastid and mitochondrial genomes from metagenomic data. This is particularly relevant for the exploration of some specific target sequences from largely contaminated datasets such as paleometagenomic samples. Profile HMM seeds can also be used to reconstruct specific protein coding genes for gene diversity studies, thus determining all possible gene variants present in a metagenomic sample, independently of their organism of origin. Such surveys could be useful to detect the emergence of drug-resistant variants in sensitive environments such as hospitals and animal production facilities, where antibiotics are regularly used. In addition, the extra length obtained with iterative progressive assembly of these target-specific sequences could reveal their genomic context, that is, whether they are originated from chromosomal or episomal sources, and surrounded by other genes involved with drug-resistance and/or associated with transposable elements. By using multiple profile HMM seeds, built from proteins from a specific pathway, GenSeed-HMM allows one to assess the occurrence of this pathway in specific environmental metagenomic samples, even if the gene complement is derived from multiple organism sources. Finally, another interesting application is the use of the progressive assembly method as an adjunct for gap closure on assembly finishing projects, by using multiple contig ends as anchored seeds to promote a sequence walking/progressive assembly process in which overlapping sequences can lead to gap closure. Using an in-house script for this specific application, we were able to close around 80% of the gaps of a bacterial sequencing project (data not shown). Concluding, GenSeed-HMM is a multipurpose program under active development, and we envisage its growing application on a variety of forthcoming projects.

## Author contributions

AG and AR conceived and designed the study. AMD, JA, and PZ contributed to the design of the experiments. AG, AR, ALO, JMG, JA, LO, MT, and TS performed the experiments. AG, AR, ALO, JMG, JA, and TS analyzed the data. DM and EM collected raw sewage samples and generated sequencing data. AG and AR prepared the first draft of the manuscript. JA and PZ participated in the discussion and writing of the manuscript. All authors revised the manuscript and have agreed to the final content.

### Conflict of interest statement

The authors declare that the research was conducted in the absence of any commercial or financial relationships that could be construed as a potential conflict of interest.
