# Supplementary material for: GenSeed-HMM: A Tool for Progressive Assembly Using Profile HMMs as Seeds and its Application in Alpavirinae Viral Discovery from Metagenomic Data
Source: Front Microbiol. 2016 Mar 4;7:269. doi: 10.3389/fmicb.2016.00269 (PMC4777721; doi:10.3389/fmicb.2016.00269)
Supplement: Supplementary file 3 [file DataSheet3.docx]

Supplementary Material

**GenSeed-HMM: A tool for progressive assembly using profile HMMs as seeds and its application in *Alpavirinae* viral discovery from metagenomic data**

**João M. P. Alves^1^**^†^**, André L. de Oliveira^1^**^†^**, Tatiana O. M. Sandberg^1^, Jaime L. Moreno-Gallego^2^, Marcelo A. F. de Toledo^1^, Elisabeth M. M. de Moura^3^, Liliane S. Oliveira^4^, Alan M. Durham^4^, Dolores U. Mehnert^3^, Paolo M. de A. Zanotto^3^, Alejandro Reyes^5,6*^ and Arthur Gruber^1*^**

^1^Department of Parasitology, Institute of Biomedical Sciences, Universidade de São Paulo, São Paulo SP, Brazil

^2^Graduate program in Computational Biology, Universidad de los Andes, Bogotá, Colombia

^3^Department of Microbiology, Institute of Biomedical Sciences, Universidade de São Paulo, São Paulo SP, Brazil

^4^Department of Computer Sciences, Institute of Mathematics and Statistics, Universidade de São Paulo, São Paulo, Brazil

^5^Department of Biological Sciences, Universidad de los Andes, Bogotá, Colombia

^6^Center for Genome Sciences and Systems Biology, Department of Pathology and Immunology, Washington University in Saint Louis, USA

***Correspondence:** Alejandro Reyes, Department of Biological Sciences, Universidad de los Andes, Carrera 1 No. 18 A – 10, Off. A307, Bogotá, Colombia. [a.reyes@uniandes.edu.co](mailto:a.reyes@uniandes.edu.co); Arthur Gruber, Department of Parasitology, Institute of Biomedical Sciences, Universidade de São Paulo, Av. Prof. Lineu Prestes 1374, São Paulo SP, 05508-000, Brazil. [argruber@usp.br](mailto:argruber@usp.br)


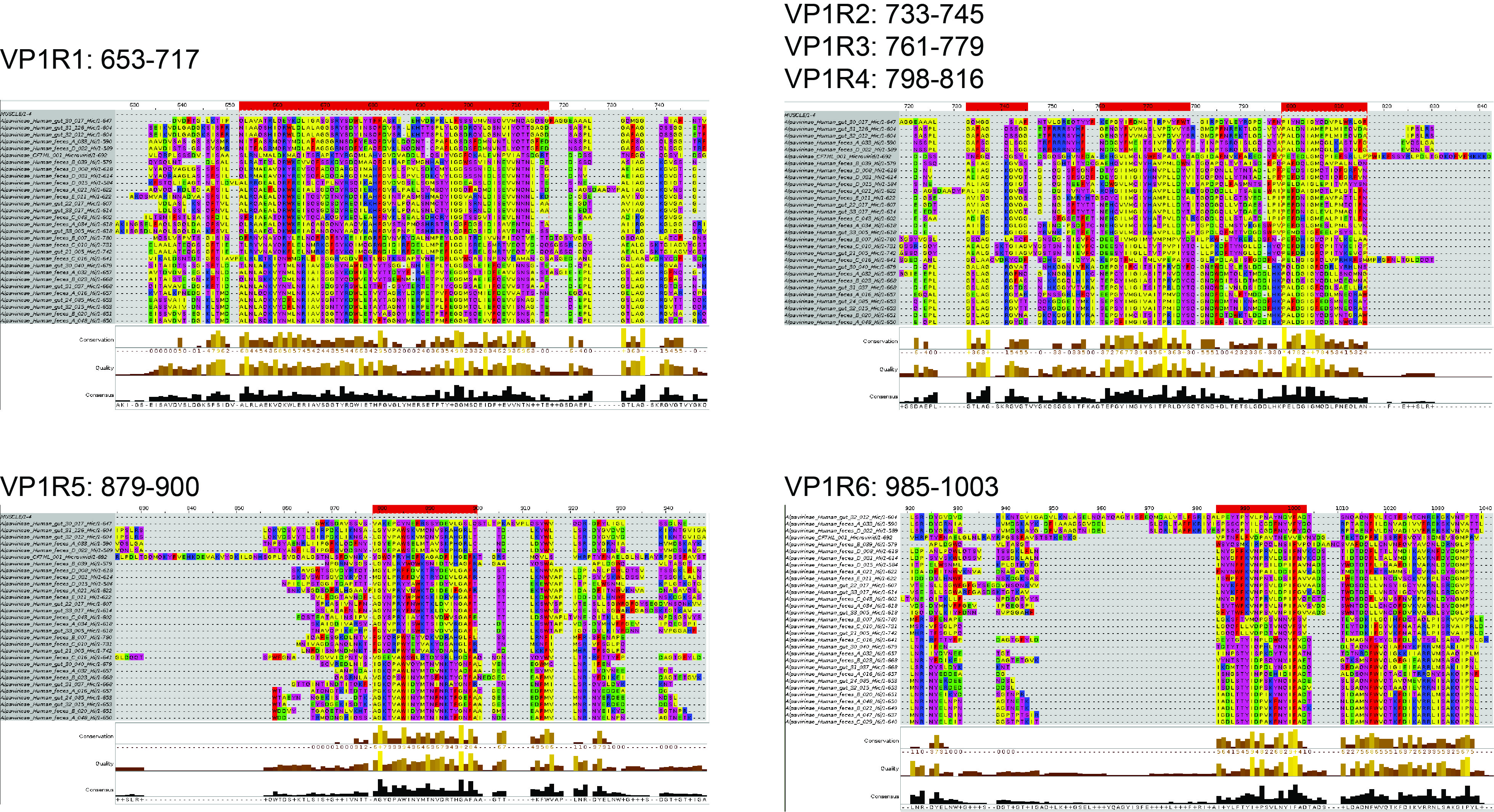


**Supplementary Figure 1: Multiple sequence alignment of VP1 proteins.** Aligned sequences were derived from reference *Microviridae* genomes (Roux et al., 2012). Four distinct conserved regions (upper red bars) were selected for profile HMM construction using *hmmbuild* (Eddy, 2011). Profile HMMs VP1R1 and VP1R4 were used for progressive assembly along with VP4R1 and VP4R3 (not shown).

A)

B)

**Supplementary Figure 2: Progressive assembly using different HMM seeds and a metagenomic sewage sample.** Contig size distribution profiles obtained by progressive assembly with GenSeed-HMM using an Illumina dataset from a sewage treatment plant at the municipality of Taboão da Serra, São Paulo, Brazil (unpublished data) and profile HMM seeds derived from *Alpavirinae* capsid proteins VP1 **(A)** and VP4 **(B)**. Contigs are ranked in decreasing order of size.


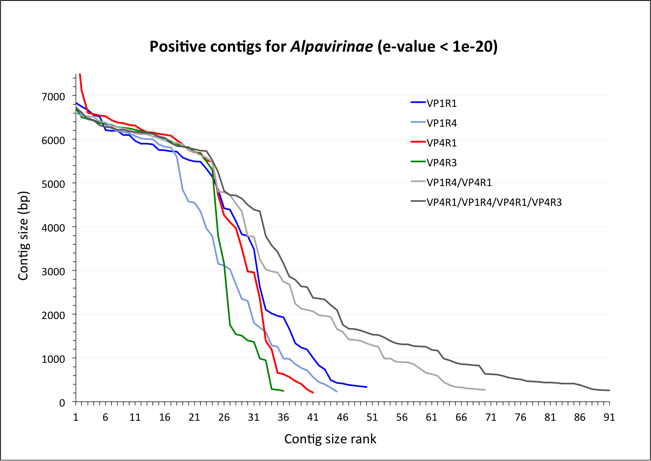


**Supplementary Figure 3: Progressive assembly using single and multiple HMM seeds.** Contig size distribution profiles obtained by progressive assembly with GenSeed-HMM using a 454 dataset from fecal samples from human patients (Reyes et al., 2010) and profile HMM seeds derived from *Alpavirinae* capsid proteins VP1 and VP4. Assemblies were performed using single or multiple seeds, as indicated. Assembled contigs were compared with *blastx* against all reference *Microviridae* proteins (Roux et al., 2012) with a cut-off E-value of 1e-20 and only contigs positive for *Alpavirinae* were selected. Contigs are ranked in decreasing order of size.

**Supplementary Table 1: Genbank entries of VP1 proteins from *Gokushovirinae* and *Microvirus* used for phylogenetic analysis.**

| **GenBank accession code** | **Description** | **Viral Subfamily** |
| --- | --- | --- |
| NP_063895.1 | hypothetical protein [*Chlamydia pneumoniae* phage CPAR39] | *Gokushovirinae* |
| NP_510872.1 | hypothetical protein PhiCPG1p2 [Guinea pig *Chlamydia* phage] | *Gokushovirinae* |
| NP_054647.1 | structural protein [*Chlamydia* phage 2] | *Gokushovirinae* |
| NP_044312.1 | VP1 [*Chlamydia* phage 1] | *Gokushovirinae* |
| NP_073538.1 | major capsid protein [*Bdellovibrio* phage phiMH2K] | *Gokushovirinae* |
| NP_598320.1 | capsid protein [*Spiroplasma* phage 4] | *Gokushovirinae* |
| NP_040678.1 | major coat protein [*Enterobacteria* phage G4 sensu lato] | *Microvirus* (genus) |
| NP_039597.1 | major coat protein [*Enterobacteria* phage alpha3] | *Microvirus* (genus) |
| YP_512416.1 | gpF [*Enterobacteria* phage WA13 sensu lato] | *Microvirus* (genus) |
| YP_512796.1 | gpF [*Enterobacteria* phage ID18 sensu lato] | *Microvirus* (genus) |
| YP_002985212.1 | gpF [*Enterobacteria* phage St-1] | *Microvirus* (genus) |
| NP_040711.1 | capsid protein [*Enterobacteria* phage phiX174 sensu lato] | *Microvirus* (genus) |

**Supplementary Table 2: Taxonomic assignment of contigs (raw sewage data, progressive assembly) and classification precision and sensitivity.** Contigs generated by GenSeed-HMM with the respective profile HMM seed (VP1R1, VP1R4, VP4R1 and VP4R3) were compared against all reference *Microviridae* proteins (Roux et al., 2012) using *blastx* with a cutoff E-value of 1e-20. Contigs were also evaluated for the presence of the VP1R4 region by *hmmsearch* and the number of positive contigs is shown.

| Subfamily | Profile HMM seed | | | | |
| --- | --- | --- | --- | --- | --- |
|  | VP1R1 | VP1R4 | VP4R1 | VP4R3 | **Total^1^** |
| *Alpavirinae* | 501 | 305 | 271 | 200 | **852** |
| *Gokushovirinae* | 237 | 0 | 243 | 216 | **462** |
| *Pichovirinae* | 159 | 1 | 81 | 85 | **198** |
| **Total** | **897** | **306** | **595** | **501** | **2480** |
| VP1R4-positive | 146 | 305 | 25 | 14 | **333** |
| Sensitivity for *Alpavirinae* | 58.80% | 35.80% | 31.81% | 23.47% |  |
| Precision for *Alpavirinae* | 55.85% | 99.67% | 45.55% | 39.92% |  |
| Sensitivity for VP1R4 | 43.84% | 91.59% | 7.51% | 4.20% |  |
| Precision for VP1R4 | 16.28% | 99.67% | 4.20% | 2.79% |  |

^1^Total number of De-replicated contigs (See Fig. 3) that belonged to a given taxonomic assignment.

**Supplementary Table 3: Detection of VP1 sequences.** Reference full-length or peptide sequences (VP1R4 region - coordinates 799-816 - see Supplementary Figure 1) from 33 *Alpavirinae* VP1 proteins (Roux et al., 2012) were submitted to *blastp* searches against 45 VP1 sequences reconstructed by GenSeed-HMM with the VP1R4 seed. Numbers of positive hits and the respective cutoff E-values used are presented.

| Protein identifier^1^ | Full-length | |  | Peptides 799-816 | |
| --- | --- | --- | --- | --- | --- |
|  | cutoff = 1e-6 | cutoff = 1e-12 |  | cutoff = 1e-2 | cutoff = 1e-6 |
| Human feces C 010 AG0199 | 29 | 10 |  | 6 | 3 |
| Human feces E 011 AG0388 | 27 | 19 |  | 13 | 1 |
| Human gut 33 005 AG0184 | 20 | 17 |  | 6 | 1 |
| Human feces D 031 AG0425 | 26 | 19 |  | 10 | 1 |
| Human feces C 043 AG0322 | 22 | 19 |  | 15 | 0 |
| Human gut 32 015 AG0213 | 23 | 14 |  | 7 | 0 |
| Human gut 21 005 AG016 | 30 | 8 |  | 6 | 3 |
| Human feces D 022 AG0392 | 20 | 5 |  | 3 | 3 |
| Human feces A 048 AG087 | 19 | 15 |  | 10 | 4 |
| Human feces C 029 AG0109 | 23 | 15 |  | 10 | 4 |
| Human gut 31 126 AG0306 | 28 | 6 |  | 3 | 0 |
| Human gut 32 012 AG0208 | 28 | 6 |  | 3 | 0 |
| Human feces A 033 AG0383 | 18 | 6 |  | 3 | 3 |
| Human gut 30 040 AG0134 | 37 | 14 |  | 8 | 2 |
| Human feces B 023 AG0145 | 22 | 15 |  | 8 | 0 |
| Human feces A 034 AG0105 | 18 | 17 |  | 13 | 1 |
| CF7ML 001 AG0309 | 16 | 8 |  | 0 | 0 |
| Human feces A 032 AG0217 | 21 | 15 |  | 12 | 0 |
| Human feces A 016 AG001 | 23 | 15 |  | 13 | 3 |
| Human gut 31 037 AG0297 | 27 | 16 |  | 13 | 0 |
| Human gut 33 017 AG0154 | 24 | 18 |  | 10 | 1 |
| Human feces D 015 AG019 | 24 | 17 |  | 3 | 1 |
| Human gut 24 085 AG0230 | 23 | 13 |  | 7 | 0 |
| Human feces B 039 AG096 | 23 | 16 |  | 7 | 1 |
| Human gut 30 017 AG0205 | 17 | 5 |  | 5 | 3 |
| Human feces A 021 AG077 | 25 | 17 |  | 11 | 0 |
| Human feces C 016 AG0274 | 19 | 10 |  | 2 | 2 |
| Human feces A 047 AG0311 | 23 | 15 |  | 10 | 4 |
| Human feces B 020 AG0351 | 27 | 15 |  | 8 | 4 |
| Human gut 22 017 AG0397 | 24 | 19 |  | 12 | 1 |
| Human feces B 021 AG0368 | 19 | 15 |  | 10 | 4 |
| Human feces B 007 AG068 | 28 | 10 |  | 5 | 2 |
| Human feces D 008 AG098 | 27 | 19 |  | 7 | 0 |
| # of unique proteins detected / average ± SD^2^ | 45 / 23±4.4 | 45 / 13±4.5 |  | 44 / 7±3.8 | 27 / 1±1.5 |

^1^As described by Roux et al. (2012).

^2^Standard Deviation.

**Supplementary Table 4: Taxonomic assignment of contigs (human fecal data, global assembly) and classification precision and sensitivity.** Contigs generated by Newbler were translated into the six frames with *transeq* and selected with the respective profile HMM (VP1R1, VP1R4, VP4R1 and VP4R3) using *hmmsearch*. The selected contigs were compared against all reference *Microviridae* proteins (Roux et al., 2012) using *blastx* with a cutoff E-value of 1e-20. Contigs were also evaluated for the presence of the VP1R4 region by *hmmsearch* and the number of positive contigs is shown.

| Subfamily | Profile HMM seed | | | | |
| --- | --- | --- | --- | --- | --- |
|  | VP1R1 | VP1R4 | VP4R1 | VP4R3 | **Total^1^** |
| *Alpavirinae* | 55 | 43 | 43 | 41 | **88** |
| *Gokushovirinae* | 15 | 0 | 14 | 16 | **23** |
| *Pichovirinae* | 1 | 0 | 1 | 1 | **1** |
| **Total** | **71** | **43** | **58** | **58** | **126** |
| VP1R4-positive | 37 | 43 | 16 | 16 | **42^2^** |
| Sensitivity for *Alpavirinae* | 62.50% | 48.86% | 48.86% | 46.59% |  |
| Precision for *Alpavirinae* | 77.46% | 100.00% | 74.14% | 70.69% |  |
| Sensitivity for VP1R4 | 88.10% | 102.38% | 38.1% | 38.1% |  |
| Precision for VP1R4 | 52.11% | 100.00% | 27.59% | 27.59% |  |

^1^Total number of de-replicated contigs (See Figure 3) that belonged to a given taxonomic assignment.

^2^As the total number of contigs is the de-replicated number, in cases where two contigs of the same seed were clustered, the total number of contigs could be less than the total number of contigs from a given seed.

**Supplementary Table 5: Taxonomic assignment of contigs (raw sewage data, global assembly) and classification precision and sensitivity.** Contigs generated by Newbler were translated into the six frames with *transeq* and selected with the respective profile HMM (VP1R1, VP1R4, VP4R1 and VP4R3) using *hmmsearch*. The selected contigs were compared against all reference *Microviridae* proteins (Roux et al., 2012) using *blastx* with a cutoff E-value of 1e-20. Contigs were also evaluated for the presence of the VP1R4 region by *hmmsearch* and the number of positive contigs is shown.

| Subfamily | Profile HMM seed | | | | |
| --- | --- | --- | --- | --- | --- |
|  | VP1R1 | VP1R4 | VP4R1 | VP4R3 | **Total^1^** |
| *Alpavirinae* | 396 | 283 | 195 | 157 | **794** |
| *Gokushovirinae* | 241 | 0 | 225 | 202 | **485** |
| *Pichovirinae* | 141 | 0 | 63 | 64 | **182** |
| **Total** | **778** | **283** | **483** | **423** | **2717** |
| VP1R4-positive | 93 | 283 | 3 | 4 | **266^2^** |
| Sensitivity for *Alpavirinae* | 49.87% | 35.64% | 24.56% | 19.77% |  |
| Precision for *Alpavirinae* | 50.90% | 100.00% | 40.37% | 37.12% |  |
| Sensitivity for VP1R4 | 34.96% | 106.39% | 1.13% | 1.50% |  |
| Precision for VP1R4 | 11.95% | 100.00% | 0.62% | 0.95% |  |

^1^Total number of de-replicated contigs (See Figure 3) that belonged to a given taxonomic assignment.

^2^As the total number of contigs is the de-replicated number, in cases where two contigs of the same seed were clustered, the total number of contigs could be less than the total number of contigs from a given seed.
